# Mapping implementation strategies to reach community-dwelling older adults in Northwest Switzerland

**DOI:** 10.1186/s13012-024-01374-8

**Published:** 2024-06-26

**Authors:** Maria Jose Mendieta, Geert Goderis, Andreas Zeller, Olivia Yip, Flaka Siqeca, Franziska Zúñiga, Leah L. Zullig, Sabina M. De Geest, Mieke Deschodt, Johan Flamaing, Suzanne Dhaini, the INSPIRE Consortium, Pia Urfer, Matthias Briel, Matthias Schwenkglenks, Penelope Vounatsou, Carlos Quinto, Eva Blozik, Katrina Obas

**Affiliations:** 1 Nursing Science, Medizinische Fakultät, Department of Public Health (DPH), Universität Basel, Bernoullistrasse 28, 4056 Basel, Switzerland; 2 Academic Centre for Nursing and Midwifery, Department of Public Health and Primary Care, KU Leuven, Louvain, Belgium; 3 Academic Center of General Practice, Department of Public Health and Primary Care, KU, Louvain, Belgium; 4 Centre for Primary Health Care, University of Basel, Basel, Switzerland; 5 Department of Population Health Sciences, Duke University School of Medicine, Durham, NC UK; 6 Center of Innovation to Accelerate Discovery and Practice Transformation, Durham Veterans Affairs Health Care System, Durham, NC UK; 7 Gerontology and Geriatrics, Department of Public Health and Primary Care, KU, Louvain, Belgium; 8 Competence Center of Nursing, University Hospitals Leuven, Louvain, Belgium; 9 Department of Geriatric Medicine, University Hospital Leuven, Louvain, Belgium; 10 University Children’s Hospital Zurich, The Eleonore Foundation, Zurich, Switzerland

**Keywords:** Implementation strategies, Implementation mapping, Reach, Fidelity, Older adults

## Abstract

**Background:**

In Northwestern Switzerland, recent legislation tackles the needs of community-dwelling older adults by creating Information and Advice Centers (IACs). IACs are a new service in the community that aims to assess the needs and provide information on age-related issues to community-dwelling older adults and their families. Previous studies reported difficulties in reaching community-dwelling older adults for community-based programs. We aimed to: 1) systematically identify implementation strategies to promote the IAC among community care providers, older adults and informal caregivers; 2) monitor the delivery of these strategies by the IAC management; and 3) describe the impact of those strategies on reach of community-dwelling older adults. This study was conducted as part of the TRANS-SENIOR project.

**Methods:**

As part of the INSPIRE feasibility assessment, we conducted a pre-test post-test study between March and September 2022. The sample included 8,840 older adults aged 65 + visiting/calling or being referred to the IAC for the first time. Implementation strategies were selected using implementation mapping and organized in bundles for each group of community care providers and older adults/caregivers. Our evaluation included: estimation of fidelity to the delivery of implementation strategies and bundles by the IAC management and their coverage; referral source of older adults to the IAC; and impact of the strategies on reach of the IAC on the 65 + population living in the care region. Adaptations to the strategies were documented using the FRAME-IS. Descriptive statistics were calculated and reported.

**Results:**

Seven implementation strategies were selected and organized in bundles for each community care provider and older adults and their caregivers. The lowest fidelity score was found in implementation strategies selected for nursing homes whereas the highest score corresponded to strategies targeting older adults and caregivers. “Informational visits” was the strategy with the lowest coverage (2.5% for nursing homes and 10.5% for hospitals and specialized clinics). The main referral sources were self-referrals and referrals by caregivers, followed by nursing homes. The IAC reach among the 65 + population was 5.4%.

**Conclusion:**

We demonstrated the use of implementation mapping to select implementation strategies to reach community-dwelling older adults. The reach was low suggesting that higher fidelity to the delivery of the strategies, and reflection on the causal pathway of the implementation strategies might be needed.

**Supplementary Information:**

The online version contains supplementary material available at 10.1186/s13012-024-01374-8.

Contributions to the literature •Reaching community-dwelling older adults is key to achieve the public health benefits of community-based programs, yet evidence from preventive programs shows that it is not optimal.

•This study shows how implementation mapping can be useful for identifying implementation strategies to reach community-dwelling older adults using public involvement.

•Although our reach was low, mainly explained by the low fidelity in delivering the implementation strategies, this study demonstrates the potential of using implementation mapping for the selection of implementation strategies to reach other vulnerable populations.

## Background

Reaching and supporting community-dwelling older adults as they age in their homes is frequently challenging. Due to the presence of multiple chronic conditions, older adults often present with complex health and social care needs, requiring multidisciplinary care and in some cases long-term support [1]. To meet these complex needs, the World Health Organization advocates the reorientation of services towards prioritizing primary care and community-based programs [2, 3]. Countries like Canada and Finland have started to shift the provision of care from hospitals to community settings [4]. However, visits of older adults to community services are not yet optimal. Examples of low reach of community-dwelling older adults mostly come from preventive programs, such as cancer screening or flu shots where less than 50% of older adults above 65 years utilize such services[5], and wait until they are very ill to demand care services [2]. Considering that reach denotes both the total number and representativeness of individuals willing to engage in a program [6], these findings highlight the need to invest efforts to maximize the reach of community-dwelling older adults.

Factors at different levels might play a role in the reach of older adults for community-based programs. For example, barriers to accessing community programs due to distance or costs [5, 7, 8], as well as physicians’ attitudes and older adults’ knowledge and beliefs towards these programs are important factors to consider [5]. In fact, existing studies about the challenges of vaccination programs for community-dwelling older adults have identified that lack of awareness of such programs and negative beliefs about adult vaccination among healthcare providers can contribute to their low reach in the community [9–11]. Similarly, in diabetes prevention program implementation, problems in reaching older adults in the community have also been described [12, 13]. However, implementation researchers of these programs found that after increasing the awareness of the program among healthcare providers, a positive impact in the referral and reach of older adults was observed [12, 13]. This highlights the influence that community healthcare providers can have in facilitating the access of older adults to targeted programs, while also denoting opportunities for improving reach through their involvement.

In 2009, Proctor and colleagues introduced the Conceptual Model of Implementation Research, which emphasizes the role of implementation strategies in facilitating the implementation of evidence-based interventions into usual care settings to achieve desired outcomes [14]. These strategies described as methods used to overcome barriers and enhance facilitators when implementing an intervention, are important for enhancing the adoption and reach of evidence-based interventions [15, 16]. Thus, strategic and targeted utilization of implementation strategies could significantly improve the reach of community-based programs, consequently leading to improved outcomes for older adults.

To date, we are not aware of recommendations on which strategies are more effective to reach community-dwelling older adults. Likewise, there is no guidance on how to select strategies to involve community care providers to reach community-dwelling older adults based on theory [17]. A case study by Puffer and Ayuku (2022) reported the use of implementation strategies to reach hard-to-reach populations for a community-embedded program for mental health [18]. However, one of the challenges described was the selection of appropriate implementation strategies to match the barriers that arose with the implementation of the evidence-based intervention and the evaluation of its effectiveness [18]. Therefore, to obtain the public health benefits of community-based programs for older adults living in the community, efforts are needed to systematically select implementation strategies to reach them, by also involving community care providers. Additionally, it is crucial to utilise standardised definitions when describing these implementation strategies. The Expert Recommendations for Implementing Change (ERIC) [15] provides a valuable framework for this. Adopting a standardised terminology not only enhances clarity but also facilitates scientific or real-world replication, by providing crucial details of the implementation strategies used to reach community-dwelling older adults.

In northwestern Switzerland, Canton Basel-Landschaft (BL) passed a new care legislation to address the needs of community-dwelling older adults and ensure high quality of care provision [19]. According to this law, the municipalities of the Canton were to be regrouped in care regions, and establish an Information and Advice Center (IAC). These centers would provide information about ageing to community-dwelling older adults and their caregivers, as well as identify the older adults’ care needs [19]. Each care region was free to decide whether to delegate the provision of this service to another care organization within the care region or to establish a center of its own. IACs would constitute a new service within the community, with no associated cost for the service users.

This legal framework set a strong foundation for establishing a partnership between the University of Basel and the Canton BL to collaborate in the co-development, implementation and evaluation of an integrated care model for community-dwelling older adults to be implemented in the IAC [20]. We conducted a contextual analysis of the Canton prior to the development of the care model to identify barriers and facilitators for the implementation of the care model [20]. Our findings suggested the need to select and implement strategies to promote the new center among community care providers and the general public in order to reach community-dwelling older adults. Therefore, our aims are to: 1) systematically identify implementation strategies to promote the IAC among community care providers, older adults and informal caregivers; 2) monitor the delivery of these strategies by the IAC management; and 3) describe the impact of those strategies on the reach of community-dwelling older adults.

## Methods

This study is embedded in the INSPIRE (ImplemeNtation of a community-baSed care Program for home dwelling senIoR citizEns) study (https://inspire-bl.unibas.ch/). INSPIRE is an implementation science project designed following the recommendations of the Medical Research Council Framework for the development and evaluation of complex interventions [21]. This multiphase study aims to develop (phase 1), as well as to assess the feasibility (phase 2) and effectiveness (phase 3) of a community-based integrated care model (the INSPIRE integrated care model) for community-dwelling older adults in one IAC [20, 22–25]. The current study is a sub-study of the feasibility assessment of the INSPIRE integrated care model to be implemented in the newly established IAC in one care region of canton BL (phase 2).

This study follows the STARI reporting standards for Reporting Implementation Studies [26].

### Study design

**Fig. 1:**
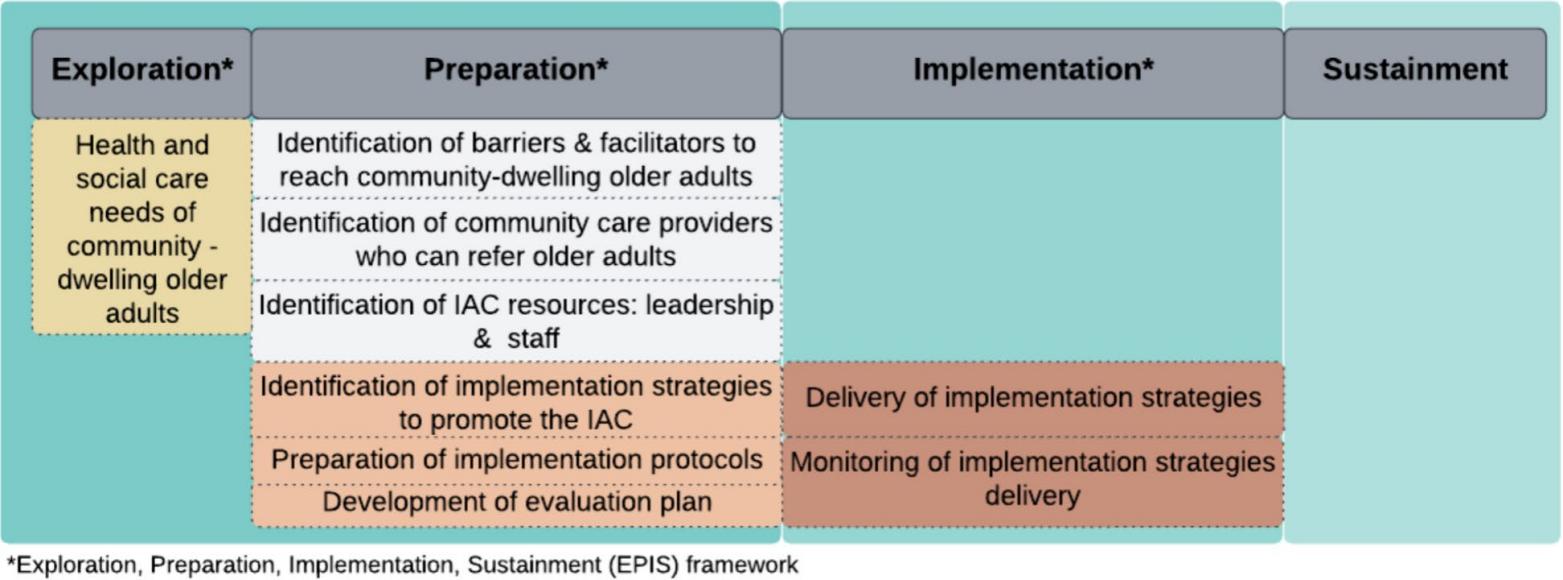
INSPIRE implementation process phases

This is a pre-test post-teststudy conducted between March and September 2022, during the preparation and implementation phases of the INSPIRE project (Fig. 1) guided by the Exploration, Preparation, Implementation and Sustainment Framework [27]. The study was conducted at the IAC of one of the ten newly established care regions of Canton BL. The protocol of the INSPIRE feasibility study has been reported [24].

### Sample & setting

Canton BL is one of Switzerland's 26 cantons. Despite its rural character, Canton BL has a growing industry, with numerous international corporations as well as small and medium-sized enterprises in sectors such as trade, construction, chemical, and pharmaceuticals [28, 29]. Its gross domestic product is 73,000 Swiss Francs per capita [29]. The care region of Canton BL, where this study took place, comprises a total population of 37,247 inhabitants, of whom 8,840 are older adults 65 + [30]. They represent 25% of the total population of the care region. Of these, 45% were older than 75 years. This care region consists of six municipalities, which collectively offer a diverse range of health and social care services for older adults. These include family physicians, nursing homes, public and private home care services, and organisations that provide companionship services [31]. However, these services are not coordinated around the needs of the older person by any health professional or care provider.

The IAC of this care region opened its doors on January 2022, financed by the contributions of the six municipalities that are part of this care region. It employs a manager (0.8 FTE), an administrative assistant (0.9 FTE), a geriatric nurse expert with 20 years of geriatric expertise who is currently a member of the research team (0.4 FTE) and a social worker (0.8 FTE). The main goals of the IAC are to provide general information about age-related topics, social and financial advice, conduct needs assessment and provide nursing home referrals when necessary, at no cost for the older person or their caregivers. Older adults could access to these services via referrals from community care providers, relatives, or themselves. The services of the IAC could be provided at the center, by phone or at the home of the older person.

All adults 65 years or more, visiting/calling or being referred for the first time to the IAC of this care region between January to September 2022 were included in the current study.

### Selection process of implementation strategies to reach community-dwelling older adults

For the identification and selection of implementation strategies to promote the IAC, we used implementation mapping recommended by Fernandez and colleagues [32]. Table 1 summarizes the five steps of implementation mapping while the next section describes its application in the current study.

**Table 1:** Implementation mapping steps according to Fernandez and colleagues

**Steps**	**Definition by Fernandez and colleagues** [26]
1. **Conduction of an implementation needs assessment**	Identification of barriers and facilitators for implementation, as well as of relevant members of the public at different levels who are crucial for the adoption, implementation and sustainment of an intervention.
2. **Identification of implementation outcomes, performance objectives, personal determinants, and development of matrices of change objectives**	Implementation outcomes or indicators for the effectiveness of implementation strategies. Performance objectives refers to tasks/behaviors required for each member of the public in order to adopt, implement or sustain an intervention. Personal determinants are modifiable factors internal to members of the public that might influence the adoption and implementation of an intervention. Development of matrices of change objectives to register the discrete changes required in each determinant in order to achieve the performance objective.
3. **Selection of theory-based methods and implementation strategies to operationalize these methods**	Theory-based methods are techniques to influence the determinants identified in step 2. Multiple methods can address one determinant and a method can influence various determinants. To operationalize these methods, implementation strategies need to be selected.
4. **Production of implementation protocols and materials**	Production of protocols for each implementation strategy. These protocols may include the purpose of the material, audience, targeted determinants and change objectives, theoretical methods or draft content.
5. **Evaluation of implementation outcomes**	Development of a plan to evaluate whether the implementation strategies have led to intended implementation outcomes and the specific implementation actions needed to implement the intervention (performance objectives).

#### Step 1- Conduction of an implementation needs assessment

In the current study, the barriers and facilitators to reach community-dwelling older adults were identified as part of the contextual analysis conducted by the research team in the preparatory phase of this project [20] (see Fig. 1). Using Stange and Glasgow’s approach to identify, analyze and report on contextual factors [33] and Pfadenhauer’s CICI framework to identify which contextual domains to consider for the analysis [34], we gathered contextual data from different sources. These sources included: cantonal and local meetings with members of the public, a cross-sectional survey, the INSPIRE Population Survey [22] and local, national and international reports [20]. As the context is a dynamic structure that evolves over time [34], additional information about the setting was collected during the implementation phase through informal conversations with the IAC staff. This activity led to the identification of new barriers. Table 2 summarizes the identified barriers and facilitators at meso and micro level that could impact the reach of the target population by the IAC.

**Table 2:** Barriers and facilitators for the implementation of an IAC in a care region of Canton BL

**Barriers**	
*Preparation phase*	
Political and legal context	Meso-level: • The care law does not specify how community care providers will collaborate with the IAC, thus there is no obligation for them to refer older adults to the IAC.
Socio-cultural context	Meso-level: • Conflicting interests of all actors involved in care, leading to different attitudes and perceptions towards the IAC services. • Presence of multiple community care providers for older adults with different outcome expectations regarding the IAC. • Insufficient/contradictory information on services available in the care region, making older adults, caregivers and community care providers less likely to be aware of and contact the IAC.
*Implementation phase*	
Setting	Micro-level: • The IAC management leads the promotion of the IAC with low front-line engagement.
**Facilitators**	
*Preparation phase*	
Socio-cultural context	Meso-level: • A champion among the research team: a family physician, head of the department of Family Medicine of the local university who voluntarily and enthusiastically contributes with ideas to overcome the barriers and facilitate the promotion of the IAC. • The members of the community, part of the cantonal group of this project, include representatives of health and social care organizations, family physicians, nurses, social workers and other health professionals, as well as representatives of senior organizations and caregivers. They contributed in the identification of barriers and provided recommendations on strategies to reach older adults.

**Fig. 2:**
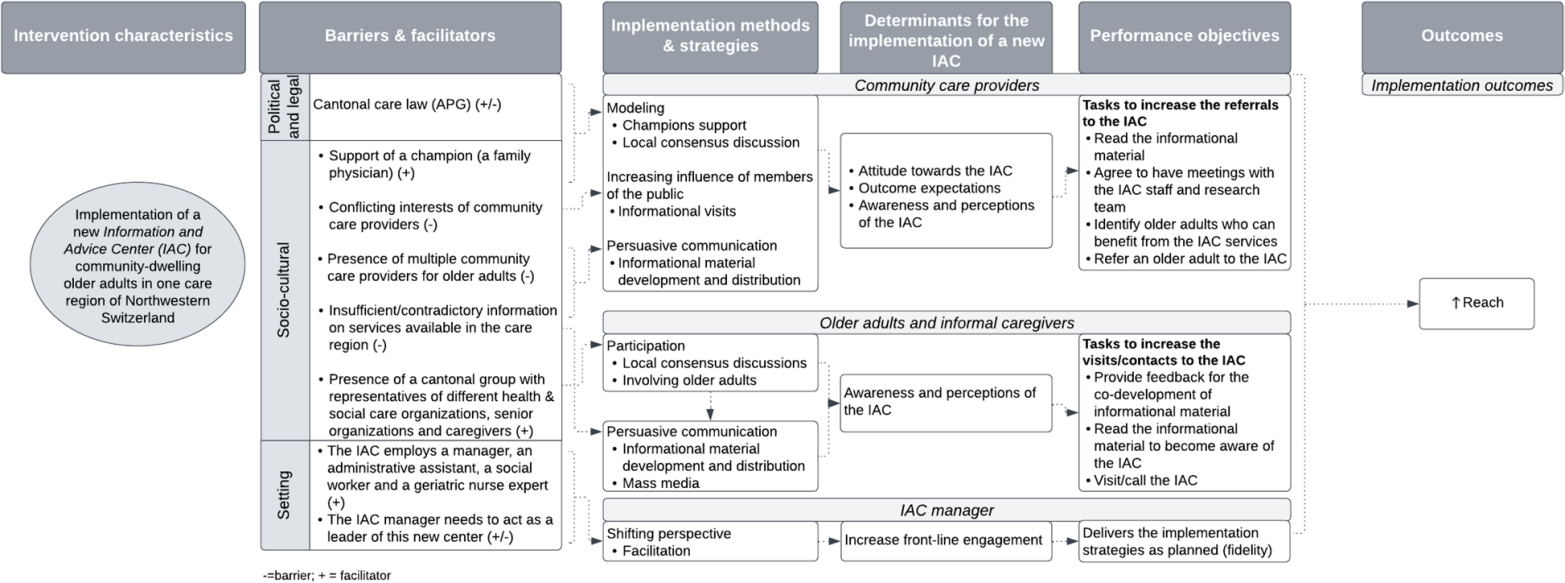
Implementation mapping logic model to reach community-dwelling older adults

Additionally, our contextual analysis allowed us to identify relevant members of the public affected by the implementation of the IAC. They were grouped in two clusters: community care providers including family physicians, heads of internal medicine departments of the main hospitals and directors of hospitals, clinics, social care organizations, nursing homes, and other organizations providing services to older adults; and older adults and their caregivers including representatives of senior organizations and community venues. In the implementation phase, we included the IAC management as a relevant member too, due to their main role in the delivery of the implementation strategies to community care providers and the older adults and informal caregivers (see Fig. 2).

#### Step 2—Identification of implementation outcomes, performance objectives, personal determinants, and development of matrices of change objectives

We selected *reach* as the implementation outcome to measure the impact of our implementation strategies, as we wanted to capture older adults who were referred, called or visited the IAC as a result of the implementation strategies delivered by the IAC management.

In parallel, we identified that to stimulate referrals, visits and calls to the IAC, each member of the public needed to perform certain tasks. Performance objectives for community care providers included: reading the informational material, meeting with the research team and IAC management, as well as identifying and referring older adults who can benefit from the services of the IAC. For the older adults and their caregivers, we identified participation of a senior organization representative in the co-development of messages for the informational material, which would be read by the older adults, as well as older adults’ visits/calls to the IAC as performance objectives. For the IAC management, the main task included the delivery of all the implementation strategies as planned (see Fig. 2).

Determinants influencing community care providers, older adults and their informal caregivers in their willingness to refer, visit or contact the IAC, respectively, were obtained from the previous step (Table 2, Fig. 2). For example, in our contextual analysis we identified that the care region had multiple care providers with conflicting interests and contradictory information. Thus, we reflected that community care providers’ awareness, perception and attitude towards the IAC or their outcome expectations about the services of the IAC may influence their decision to refer older adults to the IAC. Likewise, for older adults and caregivers, their awareness and perception of the IAC could influence their decision to contact the center. Additionally, due to the importance of the IAC management to take a leadership role in the IAC promotion*,* we identified that their level of front-line engagement could impact the overall promotion of the IAC among community care providers, older adults and caregivers (see Fig. 2). Finally, we developed a matrix of change objectives (see supplementary material A).

#### Step 3—Selection of theory-based methods and implementation strategies to operationalize these methods

We identified theory-based methods using Kok’s Intervention Mapping taxonomy of behavior change methods [35]. This taxonomy for intervention development describes behavior change methods which carefully match specific determinants (e.g., attitude) and provide practical applications [35]. In parallel, we used the Expert Recommendations for Implementing Change (ERIC) to describe these methods through several implementation strategies [15]. As multiple methods and strategies can be used to address one determinant, we created bundles of implementation strategies for each group of community care providers and older adults and caregivers. For example: separate bundles of two implementation strategies were created for hospitals and specialized clinics (e.g. a bundle includes more than one implementation strategy such as Informational material and informational visits) as well as for older adults and their caregivers (e.g. Informational material and use of mass media). Most of the strategies were selected and developed in the preparation phase. In the implementation phase, an additional implementation strategy was introduced to support the IAC management (see Table 3).

**Table 3:** Selected implementation strategies and their implementation protocol

**Implementation strategy**^**a**^ [14]	**Actor**	**Action**	**Action target**	**Temporality**	**Dose**	**Justification**
*Involving older adults*	A senior organization representative	Provides feedback, proposes changes and makes suggestions on content of the message to be included in the letter and the video, and best strategies to reach older adults in their homes.	Get feedback from patient/consumers about the messages and implementation strategies selected.	Preparation phase	Two meetings	Research has shown positive benefits of involving public and patients in the recruitment process [35].
*Champion support*	A champion, member of the INSPIRE project	Promotes the implementation of the IAC and its services among his colleagues (mostly family doctors and internal medicine specialists), through e-mails. Develops the content of letters for his colleagues and signs them Provides feedback on the script and participates as a spokesman in a video (video A). The video is inserted as a QR code in letters.	Overcome resistance in the opening of the IAC among health care professionals and increase awareness of the IAC.	Preparation phase	One e-mail sent to the heads of the internal medicine Department of 5 local hospitals One letter per family physician before and after the opening of the IAC.	Studies have reported the identification of champions as a key factor for implementation success [36].
*Local consensus discussions*	A champion and a senior organization representative	Reflect and agree on the messages on the content of the informational material to inform community care providers, older adults and caregivers and the public in general about the new IAC.	Determine the feasibility of the implementation strategies, and appropriateness of the content of the informational material.	Preparation phase	One meeting with the family physician and two meetings with the senior organization representative.	Research has shown positive benefits of involving public and patients in the recruitment process [35].
*Informational visits*	IAC management	Organizes individual meetings with targeted community care providers to raise awareness, introduce the IAC with the intent to clarify expectations regarding the opening and services offered in the center and their role in the implementation.	Knowledge about the IAC, intention to refer older adults to the IAC.	Implementation phase	One visit per targeted care provider for approximately 60 min before and after the opening of the IAC.	Research has shown positive benefits of involving public and patients in the recruitment process [35].
*Informational material development and distribution*	Three research team members, our champion, a senior organization representative and the IAC management *Developed the material* IAC management *Distributed the material*	Develop layout and final content of the letters, brochures and flyers to be sent to older adults and e-mails to be sent to community care providers Develop the script for a video (video B) for other community care providers. The video is inserted as a QR code in the e-mails. The IAC management participates as a spokesman in this video, using the local language and dialect (Swiss German). Disseminates relevant informational material according to protocol: - Community care providers: e-mails - Older adults: letters, brochures & sent by post to their homes - Older adults: flyers delivered in their homes & also through care organizations (e.g., meals on wheels)	Inform older adults & community care providers about the IAC; engage community care providers to collaborate in the referral of older adults to the IAC.	Implementation phase	One letter & brochure/flyer per older adult before and after the opening of the IAC. One e-mail per community care provider before and after the opening of the IAC.	Research has shown that informing and training members of the public can increase their involvement [37].
*Use mass media*	IAC management	- Publishes articles in the local newspaper to announce the opening date & services of the IAC. - Publishes the contact details of the IAC in the local newspaper. - Gives at least one interview in local radio.	Inform older adults & improve contact with the IAC.	Implementation phase	Ten articles & 34 biweekly announcements	Research has shown that most populations become aware of new interventions through mass media [38].
*Facilitation*	A member of the research team	Supports the IAC management to recognize the importance of engaging community care providers in the referral process of the target population.	Increases front-line engagement of the IAC management.	Implementation phase	As required	Leadership in healthcare is challenging and appropriate training is recommended [39].

^a^A refined compilation of implementation strategies: Results from the Expert Recommendations for Implementing Change (ERIC) project

#### Step 4 – Production of implementation protocols

We developed implementation protocols guided by the recommendations of Proctor and colleagues to specify implementation strategies [16]. In the implementation protocols, we described for each implementation strategy the actor (who enacts the strategy), action (specific activities that need to be enacted), action target (mechanism through which the strategy attempts to impact the implementation outcome), temporality (when the strategy is used), dose (dosage of the strategy), and the justification (theoretical justification for the selection of the strategy) [16] (Table 3).

Facilitators identified in our contextual analysis were also considered for the development of the protocols and materials. For example, a champion (member of the research team) and a Senior Organization representative were involved in the development and implementation of some of the strategies. The implementation of the selected strategies was planned to be led by the IAC management (see Fig. 2 and Table 3).

#### Step 5—Evaluation of implementation outcomes

Our evaluation plan consisted of determining the impact of the implementation strategies on reach (implementation outcome). Additionally, we included as performance objective measurements for the IAC management, the fidelity to the delivery of the implementation strategies and bundles according to the protocol and the coverage of each strategy. For community care providers, older adults and caregivers, referral source of community-dwelling older adults to the IAC was used as a performance measurement. Adaptations to the strategies were also documented.

A logic model to illustrate steps 1 to 5, and how they relate to each other is presented in Fig. 2.

#### Data sources

We captured data on the delivery of the implementation strategies to promote the IAC, adaptations and data on older adults’ reach retrospectively from January 4th to March 20th, 2022, and prospectively from March 21st to September 2022.

We used two data sources: 1) notes from informal exchange and meetings held with the IAC management to capture fidelity in the delivery and adaptations of the selected implementation strategies; and 2) IAC administrative data about the number of referrals/visitors of the IAC and the source of referral.

### Variables and measurements

#### Performance objective measurements

##### Fidelity

We calculated the fidelity to implementation strategies and corresponding bundles that were delivered by the IAC manager in the implementation phase. For each implementation strategy, a fidelity score was determined as the total number of strategy-associated activities delivered by the IAC management divided by the total number of planned activities. Using these scores, a mean score was calculated to determine the fidelity in the delivery of each bundle of implementation strategies. To track the delivery of the strategy-associated activities by the IAC management and compare them to the original plan, we developed a tracking system following the guide of Stewart et al [36]. This system included: information describing the target member of the public; implementation strategies; activities associated to each strategy; dose and frequency (planned and delivered); and number of individuals receiving the strategy in two time points (T1 = before the opening of the IAC and T2 = after the opening of the IAC). A score of 1 was given when the activity was completed as planned and 0 if it was not.

Following the recommendation of Caroll et al. (2007), we tracked the coverage of each implementation strategy by documenting the number of members of the public who received the strategy on two time points. Coverage was determined by looking at the total number of members of the public who received each activity compared to the number of targeted populations for each strategy (see supplementary material C).

*Source of referral*: consisted of the person who contacted the IAC to refer an older adult. The sources were classified into five categories: self-referred, informal caregiver, nursing home, hospital, home care organizations, family physicians and community venues. Some older adults had two referral sources (e.g., family physician and informal caregiver). For the current study, only the first referral source, as it appeared on the administrative records, was considered for the calculations. This decision was made as only in 4 clients a second referral source was reported. However, upon requesting further clarification from the IAC staff, it was confirmed that the "second referral source" included in the administrative records referred to the main contact person for the client.

#### Outcome variable

*Reach* was determined as the combined impact of the implementation strategies delivered to community care providers, older adults and their caregivers. It was measured by dividing the number of older adults 65 + who visited/called or were referred to the IAC of the care region included in this study by the total number of older adults of 65 + living in this care region. The total population of the care region included in this study was calculated using data of the INSPIRE Population Survey which was conducted in 2019 as part of the context analysis of the INSPIRE project and published elsewhere [22, 25].

#### Data analysis

We calculated descriptive statistics including frequencies and percentages. The IAC visitors were categorized into two age groups: 65–74 and 75 + years, as differences in the physical, cognitive, and psychosocial status among these two groups and their use of healthcare services have been reported [37, 38]. All analyses were conducted using R version 4.0.4 [39].

#### Adaptations to implementation strategies

To document any adaptations done to the implementation strategies during both the preparation and implementation phases, we used the Framework for Reporting Adaptations and Modifications to Evidence-based Implementation Strategies (FRAME-IS) [40]. The FRAME-IS is a tool to document modifications to implementation strategies [40].

## Results

### Selected Implementation strategies and corresponding protocols for promoting the IAC

These results address the findings of steps 3 and 4 of the implementation mapping approach guided by Fernandez [32]. We identified seven implementation strategies and developed their protocol. Table 3 summarizes these strategies, described according to the recommendations of Proctor et al. (2013) [16]. In Table 4, we then describe seven bundles of implementation strategies targeting each member of the community.

**Table 4:** Fidelity to the delivery of implementation strategies to promote the IAC by the IAC management

Target groups (n)	Implementation strategies selected	Activities	Coverage per strategy (%)	Fidelity by IAC management to each strategy (%)	Fidelity by IAC management to each bundle (%)
Family physicians (n = 31)^a^	Informational material	Letters sent by IAC management with Video A ^b^	50.0	50	50
Hospitals and specialized clinics (n = 31)	Informational material	E-mails sent by IAC management	50	50	50
	Informational visits	Meetings organized by IAC management	10.5	50	
		Phone calls by IAC management			
Home care organizations, social care organizations & other community services (n = 77)	Informational material	E-mails sent by IAC management with Video B ^c^	19.9	60	55
		Letters sent by IAC management with Video B			
		Flyers delivered by IAC management			
	Informational visits	Meetings organized by IAC management	18.2	50	
		Phone calls done by IAC management			
Nursing homes (n = 20)	Informational material	E-mails sent by IAC management with Video B	25.0	25	25
		Letters sent by IAC management with Video B			
	Informational visits	Phone calls done by IAC management	2.5	25	
		Meetings organized by IAC management			
Older adults 65 + (n = 8840) & caregivers	Informational material	Letters sent by IAC management	50.8	60	69,3
		Brochures delivered by IAC management			
		Flyers delivered by IAC management			
	Use mass media	Interviews to the IAC management in local newspaper	N/A	78,6	
		Bi-weekly adds organized by the IAC management			
		Radio interviews done by IAC management			
Community venues ^d^ (n = 19)	Informational visits	Phone calls done by IAC management	15.8	50	50
		Meetings organized by IAC management			
	Informational material	E-mails sent by IAC management with Video B	50	50	
Seniors' organizations (n = 20)	Informational material	E-mails sent by IAC management with Video B sent	50	50	50
	Informational visits	Phone calls done by IAC management	30	50	

^a^ Numbers in () indicate the number of targeted populations in the care region included in this study. ^b^ Video A addressed to family physicians, is counted as a separate action as we tracked the number of views. ^c^ Video B addressed to other community care providers, is counted as a separate action as we tracked the number of views. ^d^ Include churches, libraries, senior’s centers, pharmacies, local coffee shops/bakeries

### Fidelity to the delivery of implementation strategies used to promote the IAC

Table 4 describes the bundles of implementation strategies and activities to promote the IAC among community care providers, older adults and their caregivers. The community care providers have been divided in target groups to facilitate the visualization of the information. Our results show that the implementation strategies targeted at nursing homes were delivered with the lowest fidelity (25% for informational material and 25% for informational visits). Higher fidelity scores were observed for strategies targeting older adults and caregivers (78.6% use of mass media and 60% informational material). Similarly, the highest fidelity score per bundle was observed in the bundle of implementation strategies delivered to older adults and their caregivers (69.3%).

Additionally, Table 4 describes the coverage of each implementation strategy according to each member of the community. We observed that “informational visits” was the strategy with the lowest coverage (2.5% for nursing homes and 10.5% for hospitals and specialized clinics), while “distribution of informational material” was the implementation strategy with the highest coverage (50% for family physicians and hospitals and specialized clinics and 50.8% for older adults and caregivers).

### Source of referral

Table 5 summarizes the number of visitors/referrals by referral source and age group (65–74 years and 75 +). We found that 81.4% and 48.1% older adults aged 65–74 years and 75 + respectively self-referred to the IAC. The next highest referral sources of the older adults were nursing homes, hospitals and home care organizations for both age groups.

**Table 5:** Number of older adults’ referrals to IAC by referral source and age category

**Referral source**	**Number of referred older adults 65–74 (%)** **N = **113	**Number of referred older adults 75 + (%)** **N = **362
Self-referral^a^	92 (81.4)	174 (48.1)
Informal caregiver	8 (7.1)	71 (19.6)
Nursing home	4 (3.5)	55 (15.2)
Hospital	4 (3.5)	35 (9.7)
Home care organizations	4 (3.5)	17 (4.7)
Family physician	1(< 1)	9 (2.5)
Community venues	0	1 (< 1)

^a^older adults reaching out to the IAC themselves

### Reach of the IAC

**Fig. 3:**
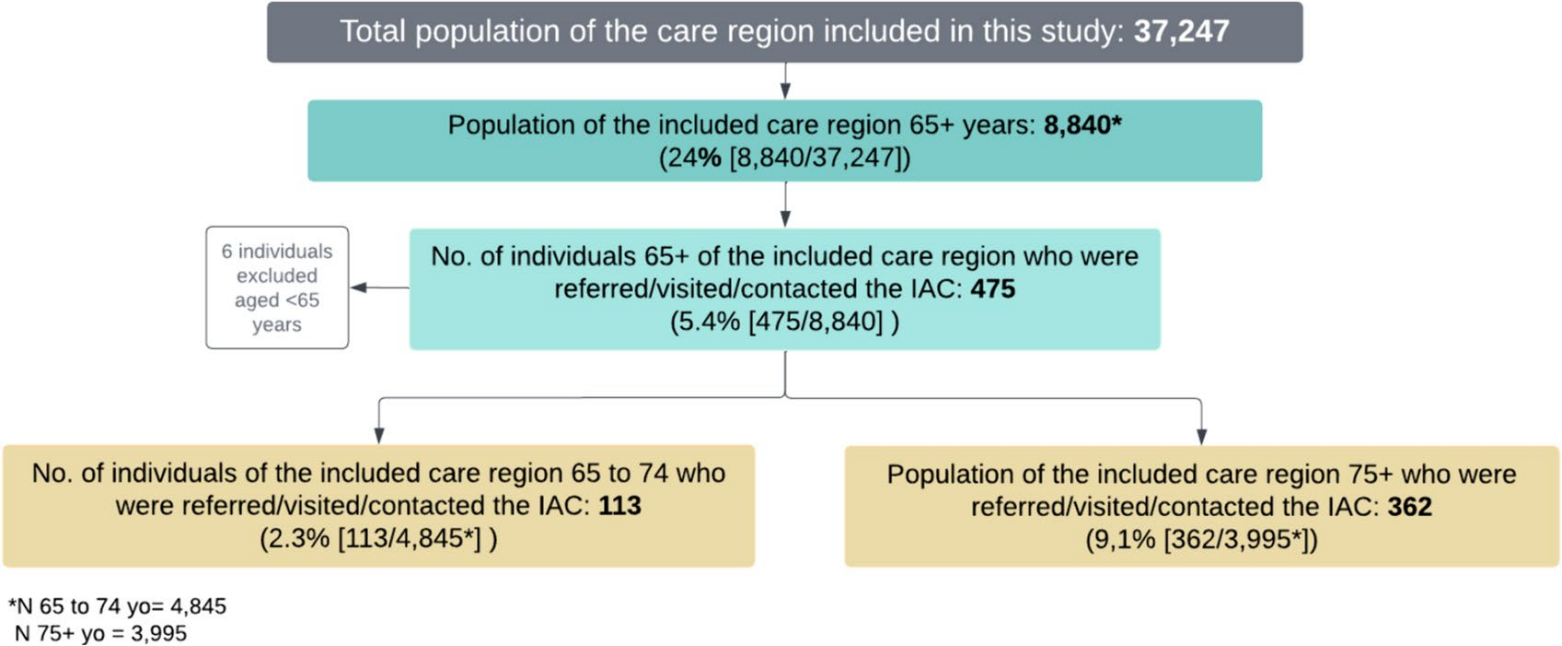
Reach of community-dwelling older adults by the IAC between January and September 2022

Figure 3 describes the impact of the implementation strategies on the reach of the IAC based on the number of referrals/visitors of the IAC. From the total number of older adults 65 + living in the care region included in this study (n = 8,840), only 5.4% were referred, visited or contacted the IAC. Among the 75 + population, the reach was 9.1%.

### Adaptations of the implementation strategies

Using the FRAME-IS, we documented adaptations to three implementation strategies: a) *informational visits to promote the IAC with community care organizations during the implementation phase, b) informational material development and distribution (letters and emails) sent to community care organizations and c) use of mass media.* Most of the modifications corresponded to changes in the temporality of delivery of the strategy (e.g., only prior or only after the opening of the IAC), which were a consequence of a decision of the IAC management. Other modifications were made to improve reach, address cultural norms or the restrictions due to the pandemic of Covid-19 including, making phone calls to the directors of nursing homes instead of visiting them in person. In these cases, the decisions were made jointly between the research team and the IAC management (Table 6).

**Table 6:** Adaptations to the implementation strategies according to the FRAME-IS^a^

**The EBP being implemented is:**		*Implementation of an Information and Advice Center to provide integrated care for (pre)frail community-dwelling older adults*							
**The implementation strategy being modified is:**	**The modification(s) being made were:**	**The reason(s) for the modification(s) is/are:**	**What was modified?**	**What was the goal?**	**What was the level of the rationale for the modification?**	**When was the modification initiated?**	**Was the modification planned?**	**Who participates in the decision to modify?**	**How widespread is the modification?**
Informational visits to promote the IAC with community care organizations in the preparation and Implementation phase	Temporality of the visits (only in the preparation or Implementation phase) to community care organizations.	Decision of the IAC management	Context (temporality of the visit, only one visit either pre or after opening).	Other: decision by the IAC management.	Organizational level (IAC management decided the temporality of the visits).	Preparation & Implementation phase	No	IAC management	Some hospitals, nursing homes, care organizations & senior organizations.
	Replacing some visits with phone calls.	• For safety reasons due to pandemic. • To address cultural norms.	Context (format, phone calls instead of in person visits).	• Increase reach of the EBP. • Increase the adoption of the EBP.	Practitioner level (IAC management).	Preparation phase	Yes	Researchers and IAC management	Community
	Involvement of a member of the research team in the visits.	Improve reach of community-dwelling older adults.	Context (personnel, a member of the research team participated in the visits too).	Increase reach of community-dwelling older adults.	Implementer level (research team).	Preparation phase	No	Researchers and IAC management	Some nursing homes & care organization.
Informational material development and distribution (letters & emails) sent to community care organizations and older adults	Temporality of the letters/e-mails sent (only in the preparation or Implementation phase).	Decision of the IAC management	Context (temporality of the letters/e-mails, only one e-mail or letter sent either pre or after opening).	Other: decision by the IAC management.	Organizational level (IAC management decided the temporality of the visits).	Preparation & Implementation phase	No	IAC management	Some hospitals, nursing homes, care organization, senior organizations & older adults.
	Replacing some letters with e-mails	To address cultural norms	Context (format, e-mails instead of letters).	• Increase reach of the EBP. • Increase the adoption of the EBP.	Practitioner level (IAC management)	Preparation phase	Yes	Researchers and IAC management	Community
Use mass media (radio announcements/interviews)	Not done	Decision of the IAC management	N/A	Other: decision by the IAC management.	N/A	N/A	N/A	IAC management	N/A

^a^The FRAME-IS: a framework for documenting modifications to implementation strategies in healthcare (34)

## Discussion

We systematically identified implementation strategies to promote an IAC and reach community-dwelling older adults in one care region of Northwest Switzerland after a law was established to reorganize care for older adults. Additionally, we monitored their implementation by measuring fidelity to their delivery by the IAC management, coverage and impact of the strategies on reach. We identified low fidelity in the delivery of implementation strategies targeted at nursing homes while the highest fidelity score was observed among older adults and their caregivers. This may have resulted in a somewhat lower reach of the target population by the IAC (5.4%) when comparing to other studies in Canada and the US that have calculated a reach of 12–19% of older adults for community-based interventions [12, 13].

Reaching community-dwelling older adults can be challenging but is essential for obtaining the potential public health benefits of community-based programs. A systematic selection of implementation strategies that address specific determinants has the potential to strengthen the collaboration of community care providers in the referral of community-dwelling older adults, which contributes to increasing their reach. Following an implementation mapping approach, we were able to identify different bundles of implementation strategies tailored to each member of the community (e.g., informational visits and informational material for nursing homes – see supplementary material B) which target specific determinants (e.g. attitudes and outcome expectations) influencing the referral of older adults to the IAC, and ultimately affecting the reach of the IAC. To the date, there is no other study that has used this approach for identifying and selecting implementation strategies aimed at reaching community-dwelling older adults. We therefore highly encourage the use of implementation mapping to guide the selection of implementation strategies. It allowed us to identify context-specific strategies, optimize resource allocation, promote stakeholder involvement and ultimately reach hard-to-reach populations such as community-dwelling older adults [41].

A key element of implementation mapping is the identification of different members of the community who can influence the implementation of an intervention [32]. Due to the regular contact that community care providers have with community-dwelling older adults, they constitute major actors in promoting community-based centers [42] and referring older adults to these centers. Moreover, by involving different community members in the implementation process, stronger relationships can be built [43]. In this study, we have identified which community care providers provided crucial contribution to the referral of older adults to the IAC. However, our results showed low referrals of older adults, especially by home care organizations, family physicians and community venues. A possible explanation lies in the low delivery of the implementation strategies by the IAC manager leading to low coverage, with informational visits being the strategy with the lowest coverage. Additionally, we consider that we could have overlooked important barriers and determinants (steps 1 & 2), as the contextual information used were derived from the contextual analysis of the whole Canton conducted in 2019 [20] and not specifically from the care region where this study took place. Moreover, in our evaluation plan, further performance measures of community care providers and their impact on overcoming personal determinants (attitudes towards the IAC) were not considered, nor was the link between strategies used and the mechanisms through which each implementation strategy is hypothesized to work [44]. Therefore, we recommend that the evaluation plan must start by reflecting back on the causal pathway of implementation strategies used and their outcomes to identify moderators or pre-conditions that can affect the causal mechanisms of implementation strategies, as this will help to decode why a strategy achieved its intended effect or not [45].

We acknowledge the low reach of the IAC among the target population. We reflect on potential reasons which could explain this result. *First*, problems with fidelity to the delivery of the bundles of implementation strategies impacted the level of information and awareness of the IAC among community care providers. Having an unclear notion of the role of this new center, may have impacted their decision to refer older adults to the IAC. Existing interventional studies have reported a low reach of older adults due to the lack of awareness and negative beliefs among community care providers (e.g. family physicians) towards these programs [9, 10, 12, 13]. *Second*, implementing new services in the community can be a challenge in itself: it takes time to build trusting relationships and convince care organizations, with a long history in the community, to be open to collaboration [43, 46]. Additionally, when there are no formal structures to define the degree of collaboration between a new center and different care providers, the collaboration relies mostly on informal relationships that can be lost over time [43]. In our case, the legal framework (APG) supporting the implementation of the IAC does not specify the responsibilities of community care providers and their degree of collaboration with the center [19]. This could have contributed to the lack of a shared vision on the role of the IAC, and consequently, the low level of referrals to the IAC. Thus, to improve the reach of community-dwelling older adults, major efforts are needed. For example, this can be reached by creating regulatory frameworks to improve the quality of care of community-dwelling older adults that promote the establishment of formal contracts or agreements between care providers and new community-based centers. These regulations could clarify roles, processes and responsibilities of all parties involved. Additionally, it is important to invest in efforts to promote communication, and build good relationships with care providers, so that they engage in the referral of adults to community-based centers.

Establishing good relationships and motivating care providers to collaborate effectively requires high levels of engagement at the front-line, an attribute often associated with leadership [46]. Multiple studies have highlighted the importance of leadership in healthcare, especially for populations with complex needs, such as frail older adults [43, 47, 48]. Effective leadership in this context demands essential skills such as organizational and change management abilities and a visionary perspective. This enables healthcare leaders to effectively influence others at the service level, for the benefit of populations [49]. For example, the supportive leadership of the strategic director of a hospital in Hungary was described as an enabler to persuade physicians to effectively participate in an integrated care program to improve clinical outcomes for cancer patients [43]. In the current study, we observed low levels of front-line engagement by the IAC management, reflected by the low level of delivery of the implementation strategies. This could have negatively impacted the engagement of community care providers, and ultimately could also explain the low reach of the target population.

### Strengths and limitations

Although we were able to track data on the delivery of the selected implementation strategies, we could not estimate the effectiveness of each of them. In our original plan, we aimed to collect data to identify which implementation strategies were more effective to reach older adults. However, due to cultural reasons, namely older adults’ concerns about the privacy and confidentiality of their personal information, lack of interest to participate in research, and to avoid creating additional burdens for the older adult and the staff of the IAC, we decided not to pursue this aim. Finally, as there are not previous studies that assess the fidelity of implementation strategies to reach community-dwelling older adults, we did not have a benchmark score to compare our results to.

Nevertheless, the systematic selection and reporting of implementation strategies using pre-existing frameworks used in this study offers several strengths: 1) The use of a methodical process for selecting implementation strategies ensures thorough identification and selection of context-specific strategies. This approach represents a new method that can be applied to identify strategies for reaching any hard-to-reach populations, such as community-dwelling older adults; 2) transparent reporting using established frameworks such as ERIC enhances credibility and replicability in real-world settings. Finally, as this study is part of the feasibility evaluation of the INSPIRE care model, the results obtained will help shape our strategies for reaching community-dwelling older adults for the next phase, the effectiveness evaluation. For example, our findings demonstrated that the new IAC requires strong leadership and time to establish itself and build trusting relationships in the community. Thus, to reach community-dwelling older adults of the next phase, we will collaborate with already established organizations in the community.

Despite the progress made in this study, several gaps remain to be addressed. For instance, further research could focus on evaluating the fidelity and effectiveness of the identified implementation strategies in reaching hard-to-reach populations, such as community-dwelling older adults, using longitudinal designs. Assessing the fidelity and impact of these strategies on improving access to community care services would provide valuable insights into their efficacy and potential areas for improvement. Similarly, future studies could examine the reproducibility of the approach used in this study for selecting implementation strategies in different settings and populations. By assessing the consistency and effectiveness of this approach in different settings, researchers can provide valuable insights into its applicability and scalability, ultimately informing the development of evidence-based practices for implementation science. Finally, despite the use of the definition of 'reach' according to the RE-AIM framework, we didn't assess the reasons for the older adults to (not) contact the IAC. Therefore, we believe that future research could benefit from qualitative investigation, particularly through interviews with older adults. Such approach would allow a deep understanding of the motivations and decision to contact or not contact community services.

## Conclusion

This study provides information on how to systematically select implementation strategies to reach community-dwelling older adults in order to provide health care programs delivered to the community. At the same time, we measured their implementation by considering implementation outcomes and performance objectives. The lack of fidelity observed in the delivery of the implementation strategies partially explains the low reach of community-dwelling older adults. As other factors could explain the low reach of the target population, there might be the need to reflect back on the causal pathway of the implementation strategies and the related outcomes. Therefore, prior to the identification of implementation strategies to engage community care providers in the referral process, in-depth contextual analysis and careful consideration of the mediators and moderators of the mechanism of action of implementation strategies is required. We believe that the results of this study can provide valuable information for implementers and community leaders on how to improve the reach of older adults living at home, so they can gain the public health benefits of new services and programs established for them in the community.

### Supplementary Information

Supplementary Material 1.

Supplementary Material 2.

## Data Availability

The dataset used in the current study (INSPIRE Feasibility Study) is not publicly available but can be requested to the corresponding author on reasonable request.

## Abbreviations

INSPIRE: ImplemeNtation of a community-baSed care Program for home dwelling senIoR citizEns
Canton BL: Canton Basel-Landschaft
IAC: Information and Advice Center
