# Supplementary material for: Mapping implementation strategies to reach community-dwelling older adults in Northwest Switzerland
Source: Implement Sci. 2024 Jun 26;19:44. doi: 10.1186/s13012-024-01374-8 (PMC11210125; doi:10.1186/s13012-024-01374-8)
Supplement: Supplementary file 2 — Supplementary Material 2. [file 13012_2024_1374_MOESM2_ESM.docx]

**Supplementary material A**

**Identification and selection of performance objectives, personal determinants, change objectives, methods and implementation strategies**

| **Target members of the public** | **Implementation outcomes** | **Performance objectives** | **Determinants** | **Change objectives** | **Methods^1^** | **Implementation strategies** |
| --- | --- | --- | --- | --- | --- | --- |
| **Preparation phase** | | | | | | |
| **COMMUNITY CARE PROVIDERS** | *Reach*  *Fidelity* | - Read the informational material to become informed about the IAC - Agree to have meetings with the IAC staff and research team - Identify older adults who can benefit from the IAC services - Contact the IAC to refer an older adult | Attitude, awareness and perceptions of the IAC | Express the importance of referring older adults to the IAC | - Persuasive communication | - Informational material development and distribution |
|  |  |  |  |  | - Modeling | - Champions support - Local consensus discussions |
|  |  |  | Outcome expectations | - Expect that the IAC services will help to improve the care of older adults compared to the current practice - Expect that the quality of life of older adults will improve with the IAC services |  |  |
|  |  |  |  |  | - Increasing influence of members of the public | - Informational visits |
| **OLDER ADULTS AND INFORMAL CAREGIVERS** |  | - Provide feedback for the development of informational material - Read the informational material to become aware of the IAC - Visit/call the IAC | Awareness and perceptions of the IAC | - Describe the services of the IAC - Describe the access or communication with the IAC as not too complex | - Participation | - Local consensus discussions - Involving patients/consumers |
|  |  |  |  |  | - Persuasive communication | - Informational material development and distribution - Mass media |
|  |  | **Implementation phase** | | | | |
| **IAC MANAGEMENT** |  | - Delivers all the implementation strategies identified by the research team | Attitude (level front-line engagement) | - Establish an understanding of the role of the IAC with community care providers, by being visible through visits and messages - Express the importance of building trust with community care providers | - Shifting perspective | - Facilitation by INSPIRE team member |

^1^ Methods selected based on the taxonomy of behavior change methods of Kok G. and colleagues (29)

**Supplementary material B**

**Supplementary material C**

**Table xx. Tracking system of the delivery of implementation strategies to reach community-dwelling older adults**

| **Target groups (n)** | **Implementation strategies selected** | **Activities** | **Dose frequency** | | **Coverage** | |
| --- | --- | --- | --- | --- | --- | --- |
|  |  |  | **Dose frequency of planned activity ^b^** | **Dose frequency of delivered activity ^b^** | **n ^c^ of members of the public receiving the activity (T1)** | **n ^c^ of members of the public receiving the activity (T2)** |
| Family physicians (n=31) ^a^ | Informational material development & distribution | Letters sent by IAC management with Video A | 2 | 1 | 31 | 0 |
| Hospitals and specialized clinics (n=31) | Informational material development & distribution | E-mails sent by IAC management | 2 | 1 | 31 | 0 |
|  | Informational visits | Meetings organized by IAC management | 2 | 1 | 0 | 1 |
|  |  | Phone calls by IAC management | 2 | 1 | 12 | 0 |
| Home care organizations, social care organizations & other community services (n=77) | Informational material development & distribution | E-mails sent by IAC management with Video B | 2 | 1 | 77 | 0 |
|  |  | Letters sent by IAC management with Video B | 2 | 1 | 0 | 0 |
|  |  | Flyers delivered by IAC management | 1 | 1 | NP ^d^ | 3 |
|  | Informational visits | Meetings organized by IAC management | 2 | 1 | 0 | 17 |
|  |  | Phone calls done by IAC management | 2 | 1 | 39 | 0 |
| Nursing homes (n=20) | Informational material development & distribution | E-mails sent by IAC management with Video B | 2 | 1 | 20 | 0 |
|  |  | Letters sent by IAC management with Video B | 2 | 0 | 0 | 0 |
|  | Informational visits | Phone calls done by IAC management | 2 | 1 | 2 | 0 |
|  |  | Meetings organized by IAC management | 2 | 0 | 0 | 0 |
| Older adults 65+ (n=8840) & caregivers | Informational material development & distribution | Letters sent by IAC management | 2 | 1 | 0 | 8840 |
|  |  | Brochures delivered by IAC management | 2 | 1 | NP | 8840 |
|  |  | Flyers delivered by IAC management | 1 | 1 | NP | 200 |
|  | Use mass media | Interviews to the IAC management in local newspaper | 11 | 4 | NA | NA |
|  |  | Bi-weekly adds organized by the IAC management | 29 | 29 | NA | NA |
|  |  | Radio interviews done by IAC management | 2 | 0 | NA | NA |
| Community venues: churches, libraries, senior’s centers, pharmacies, local coffee shops/bakeries (n=19) | Informational visits | Phone calls done by IAC management | 2 | 1 | 10 | 0 |
|  |  | Meetings organized by IAC management | 2 | 1 | 0 | 2 |
|  | Informational material development & distribution | E-mails sent by IAC management with Video B | 2 | 1 | 19 | 0 |
| Seniors' organizations (n=20) | Informational material development & distribution | E-mails sent by IAC management with Video B sent | 2 | 1 | 20 | 0 |
|  | Informational visits | Phone calls done by IAC management | 2 | 1 | 12 | 0 |

^a^ Numbers in () indicate the number of targeted populations in the care region included in this study. ^b^ Planned activities refer to the activities planned by the INSPIRE team; Delivered activities refer to the activities delivered by the IAC management. ^c^ n refers to the number of individuals who received the activity in each time point (T1 & T2). ^d^ Activity was planned to be delivered only at one time point
